# A multi-algorithm prognostic model combining inflammatory indices and surgical features in distal cholangiocarcinoma

**DOI:** 10.3389/fonc.2025.1625703

**Published:** 2025-07-21

**Authors:** Yi Yin, Luyuan Bai, Xinyue Mu, Shan Zhang, Panpan Zhai

**Affiliations:** ^1^ Pediatrics Hospital, The First Affiliated Hospital of Henan University of Chinese Medicine, Zhengzhou, Henan, China; ^2^ School of Pediatrics, Henan University of Chinese Medicine, Zhengzhou, Henan, China; ^3^ Oncology Department, Henan Province Hospital of Chinese Medicine (The Second Affiliated Hospital of Henan University of Chinese Medicine), Zhengzhou, Henan, China

**Keywords:** machine learning, pancreaticoduodenectomy, distal cholangiocarcinoma, derived neutrophil-to-lymphocyte ratio (dNLR), prognosis

## Abstract

**Background:**

Derived neutrophil-to-lymphocyte ratio (dNLR) is an emerging blood-based inflammatory biomarker previously reported to have prognostic value in various malignancies. This study aimed to investigate the prognostic significance of dNLR in patients with distal cholangiocarcinoma (dCCA) after curative resection.

**Methods:**

Clinicopathological data of patients with dCCA in our hospital from Jan.2014 to Jun.2024 was analyzed retrospectively. Receiver operating characteristic (ROC) curve analysis was performed to evaluate the predictive value of dNLR and to identify the optimal cutoff. Survival differences between groups stratified by dNLR were compared using Kaplan-Meier analysis. Candidate variables were screened through univariate analysis using Kaplan-Meier, random forest, Recursive Feature Elimination (RFE) and least absolute shrinkage and selection operator (LASSO) regression models. Multivariate Cox regression analysis identified independent prognostic factors, which were subsequently integrated into a predictive model visualized via a nomogram. Model performance was assessed using ROC curves, calibration curves, and decision curve analysis (DCA).

**Results:**

A total of 177 patients were enrolled in this study. ROC analysis revealed an area under the curve (AUC) of 0.707 for dNLR in predicting postoperative survival, with an optimal cutoff value of 1.60. Patients stratified into a low-dNLR group (≤ 1.60) demonstrated significantly improved recurrence-free survival (41 months) and overall survival (17 months) compared to those in the high-dNLR group (> 1.60) (*p* < 0.05). Univariate and multivariate combined with 3 machine learning analyses identified preoperative dNLR > 1.60 as an independent adverse prognostic factor for postoperative outcomes, incorporating with other independent predictors (preoperative total bilirubin, carbohydrate antigen 19–9 levels, T-stage, portal venous system invasion, and lymph node metastasis) further enhanced the predictive accuracy of the prognostic model.

**Conclusion:**

A preoperative dNLR > 1.60 is an independent risk factor associated with poor prognosis in patients with dCCA. The clinical prediction model based on machine learning incorporating dNLR effectively predicts postoperative outcomes in this patient population.

## Introduction

Cholangiocarcinoma (CCA) represents a malignant tumor arising from biliary epithelial cells and constitutes one of the less common malignancies. Distal cholangiocarcinoma (dCCA), originating from the mid-to-lower portion of the common bile duct, accounts for approximately 30%–40% of all extrahepatic cholangiocarcinomas (1). Risk factors for dCCA notably include bile duct stones, cholestasis, chronic biliary inflammation, and anatomical abnormalities of the biliary tree (2). Due to poor responsiveness to chemotherapy, surgical resection remains the mainstay of treatment; however, early-stage dCCA is often clinically occult, leading to a generally poor prognosis (3–5). Currently, most cholangiocarcinoma research predominantly focuses on intrahepatic cholangiocarcinoma (iCCA), whereas dCCA lacks novel biomarkers for accurately predicting recurrence risk and clinical outcomes. Effective prognostication of dCCA patient survival can significantly aid clinicians in optimizing clinical decisions, individualizing follow-up intervals, and enabling earlier detection of tumor recurrence or metastasis, thereby potentially improving long-term patient outcomes.

Inflammation is now recognized as one of the critical driving factors in carcinogenesis and tumor progression, with chronic inflammatory states believed to accelerate tumor growth and adversely impact patient prognosis (6). Consequently, accurately assessing inflammatory status in cancer patients could effectively predict clinical outcomes. Blood-based inflammatory markers, routinely derived from complete blood counts, offer distinct advantages due to their easy availability and simple computation. Various indices, including the lymphocyte-to-monocyte ratio (LMR), platelet-to-lymphocyte ratio (PLR), systemic inflammatory response index (SIRI), and systemic immune-inflammation index (SII), have demonstrated prognostic value in patients with dCCA (7–10). The derived neutrophil-to-lymphocyte ratio (dNLR), which integrates counts of peripheral leukocytes and neutrophils, was initially introduced by Proctor et al. in 2012, demonstrating prognostic efficacy comparable to the conventional neutrophil-to-lymphocyte ratio (NLR) across multiple cancer types (11). Subsequently, dNLR has been widely adopted as a prognostic indicator in various malignancies, including gastric, pancreatic, and breast cancers, where an elevated dNLR is consistently associated with poor outcomes (12–14).

Within biliary malignancies, dNLR has also demonstrated considerable prognostic utility. Grenader et al. reported, in a cohort of 462 advanced cholangiocarcinoma patients, that low dNLR was significantly associated with improved overall survival and better responsiveness to gemcitabine-cisplatin chemotherapy compared with high dNLR (15). This finding was further supported by Buyuksimsek et al., who validated prognostic significance of dNLR in predicting outcomes for advanced CCA patients treated with gemcitabine plus oxaliplatin (GEMOX) (16). Additionally, Zhang et al. demonstrated, in a retrospective analysis of 231 surgically treated iCCA patients, that elevated preoperative dNLR strongly correlated with reduced recurrence-free survival and overall survival (17). However, despite these advances, no prior studies have assessed the prognostic relevance of dNLR specifically in dCCA patients following curative resection.

Therefore, the present study aimed to retrospectively evaluate patients with dCCA who underwent curative surgery at our center, focusing specifically on elucidating the prognostic value of preoperative dNLR. Through this investigation, we seek not only to expand the clinical utility of dNLR as a biomarker but also to identify novel, potentially effective predictors of postoperative outcomes for dCCA patients.

## Methods

### Ethics approval and consent to participate

The study was conducted in accordance with the Declaration of Helsinki (as revised in 2013) and approved by the Ethics Committee of The First Affiliated Hospital of Henan University (No. 2024-412). Due to the retrospective nature of the study, participant informed consent was waived, and the study design was approved by the appropriate ethics review board

### Patients and clinicopathological factors

This retrospective study analyzed data from patients with dCCA who underwent surgical treatment at our institution between Jan.2014 and Jun.2024. Patients were screened based on the following inclusion and exclusion criteria. The authors are accountable for all aspects of the work in ensuring that questions related to the accuracy or integrity of any part of the work are appropriately investigated and resolved.

### Inclusion and exclusion criteria

Inclusion criteria (1): Underwent surgical resection for biliary lesions at our department between January 2014 and June 2024 (2); Preoperative assessments confirmed no contraindications for surgery (3); Postoperative pathological diagnosis of dCCA (adenocarcinoma) (4); Availability of clinical data and complete follow-up information.

Exclusion criteria (1): Evidence of concurrent bacterial or viral infection preoperatively (2); Coexisting autoimmune diseases (3); History or coexistence of other malignancies (4); Receiving neoadjuvant chemotherapy before surgery (5); Perioperative mortality.

### Patient stratification and definitions

Preoperative complete blood count (CBC) data, specifically total leukocyte and neutrophil counts within one week before surgery, were extracted for each patient. The dNLR was calculated as follows: Neutrophil count/(White blood cell count - Neutrophil count). Receiver operating characteristic (ROC) curve analysis was conducted to evaluate the predictive performance of dNLR regarding one-year postoperative survival, and the area under the ROC curve (AUC) was calculated to identify the optimal dNLR cutoff value. Subsequently, patients were stratified into distinct groups based on this optimal cutoff, and comparisons were performed between these groups.

### Data collection and follow-up protocol

Baseline demographic data, intraoperative data, and postoperative recovery data were extracted from electronic medical records at our institution, and comparisons of perioperative characteristics were conducted between patient groups. Follow-up assessments were scheduled at 1 and 3 months post-discharge, every 3 months up to 2 years, and subsequently at 6-month intervals beyond the 2-year mark. Follow-ups were performed via outpatient clinic visits or telephone calls conducted by trained personnel. The primary endpoint of follow-up was patient death, and the secondary endpoint was tumor recurrence. Follow-up evaluations included blood tests (CBC, serum biochemistry, carbohydrate antiten 19-9) and radiological imaging (contrast-enhanced abdominal CT, chest CT), documentation of adjuvant treatments, and assessment of recurrence and survival status.

### Statistical analysis

Continuous variables with normal distribution were expressed as mean ± standard deviation (SD), while those with non-normal distribution were presented as median and interquartile range (IQR). Missing data were handled by following methods depending on the type of data. Patients with missing survival and tumor recurrence data and other categorized data were excluded to avoid the potential impact of data imputation. Normally distributed indexes with missing data were imputed with mean value, while non-normally distributed indexes with missing data were imputed with median value. Comparisons between groups for normally distributed continuous variables were performed using Student’s t-test, whereas non-normally distributed variables were analyzed using the Mann-Whitney U test. Categorical variables were compared using the chi-square (χ²) test or Fisher’s exact test when the sample size was ≤40 or expected frequency was <1. The “randomForestSRC”, “Recursive feature elimination” and “LASSO regression” were used to screen candidate variables and rank the importance for all factors. Survival curves were constructed using Kaplan-Meier analysis, with differences between groups assessed via the log-rank test. Independent prognostic factors were identified by multivariate analysis employing Cox proportional hazards regression. Statistical analyses were conducted with SPSS software version 26.0 and R Studio (version R 4.4.3), with a *p <*0.05 considered statistically significant. Figures were generated using R Studio (version R 4.4.3) and GraphPad Prism (version 9.0).

### Construction and validation of the prognostic nomogram

Variables identified in the multivariate analysis were cross-referenced with the top-ranked features from the random forest analysis, recursive feature elimination and the LASSO coefficients. Multiple methods were employed for variable selection in this study. Random survival forests were used to evaluate the importance of each feature. Variable importance was quantified by the increase in cumulative out-of-bag (OOB) prediction error after permutation, and node splitting was based on the log-rank statistic. The Random survival forest (RSF) model was constructed with 2000 trees. Additionally, a Cox proportional hazards model with L1 regularization (LASSO-Cox) was implemented to perform variable selection while controlling model complexity. The optimal regularization parameter (λ) was determined using 10-fold cross-validation, and variables with non-zero coefficients at the λ_min value were retained for further analysis. Recursive feature elimination was also applied to iteratively remove the least informative features. Cross-validation and hyperparameter tuning were applied to improve the model’s robustness and interpretability and avoid overfitting. SHapley Additive exPlanations (SHAP) were utilized to interpret of machine-learning models and visualize their variable importance. The overlapping factors of machine-learning models were then integrated to develop a robust survival prediction nomogram model. The predictive performance of the nomogram was evaluated using ROC curves (constructed using the “pROC” package in R). Model discrimination was assessed using the bootstrap-corrected concordance index (c-index) (“rms” package in R). Model calibration was visualized through calibration plots (“rms” package in R). The clinical utility of the nomogram was analyzed using decision curve analysis (DCA) (“ggDCA” package in R).

## Results

### Patient demographics and clinical characteristics

A total of 177 patients were included in this study, comprising 89 males and 88 females, with a median age of 66.0 (IQR: 57.0–72.0) years. Among the included patients, 90 had a history of hypertension and 46 had a history of diabetes. Jaundice was the initial symptom in 148 patients, among whom 87 received preoperative biliary drainage procedures: 19 underwent endoscopic retrograde cholangiopancreatography (ERCP) with biliary stent placement, and 68 underwent percutaneous transhepatic cholangial drainage (PTCD). Other presenting symptoms included abdominal pain (n=17) and gastrointestinal symptoms (n=5). The detailed baseline clinical characteristics are summarized in **Table 1**.

**Table 1:** Comparison of perioperative condition and major postoperative complications between low and high dNLR group.

Variables	Total (n=177)	Low dNLR group (n=53)	High dNLR group (n=124)	*P* Value
Gender [n (%)]				0.206
Male	89 (50.3)	31 (58.4)	58 (46.8)	
Female	88 (49.7)	22 (41.5)	66 (53.2)	
Age (years)	66.0 (57.0, 72.0)	66.0 (58.0, 72.0)	65.5 (57.0, 71.2)	0.928
Hypertension				0.522
Yes	90 (50.8)	25 (47.2)	65 (52.4)	
No	87 (49.2)	28 (52.8)	59 (47.6)	
Diabetes [n (%)]				0.299
Yes	46 (26.0)	11 (20.8)	35 (28.2)	
No	131 (74.0)	42 (79.2)	89 (71.8)	
BMI (kg/m^2^)	23.8 ± 3.4	23.6 ± 3.3	23.9 ± 3.5	0.621
Preoperative jaundice reduction treatment [n (%)]				0.317
Yes	87 (49.15)	23 (43.40)	64 (51.61)	
No	90 (50.85)	30 (56.60)	60 (48.39)	
Preoperative WBC count (×10^9^/L)	6.0 (5.1, 7.4)	5.5 (4.5, 6.6)	6.3 (5.3, 7.9)	0.002
Preoperative NE count (×10^9^/L)	4.0 (3.1, 5.0)	3.0 (2.4, 3.7)	4.4 (3.4, 5.7)	<0.001
Preoperative LYM count (×10^9^/L)	1.4 (1.0, 1.8)	1.8 (1.5, 2.2)	1.2 (0.9, 1.6)	<0.001
Preoperative LMR	3.3 (2.3, 4.5)	4.5 (3.7, 6.5)	2.8 (2.1, 3.8)	<0.001
Preoperative SII	669.5 (412.8, 1030.4)	355.6 (266.2, 473.6)	847.8 (597.1, 1249.8)	<0.001
Preoperative PLR	168.2 (115.8, 235.5)	114.4 (91.7, 154.6)	194.9 (142.9, 259.0)	<0.001
Preoperative ALB (g/L)	36.7 ± 5.4	37.1 ± 5.6	36.5 ± 5.4	0.542
Preoperative TB (μmol/L)	85.7 (32.1, 185.3)	49.0 (23.1, 183.0)	118.2 (50.5, 188.4)	0.023
Preoperative CEA (U/ml)	1.7 (1.1, 2.7)	1.5 (1.0, 2.0)	1.9 (1.2, 3.0)	0.013
Preoperative CA19-9 (U/ml)	63.6 (26.1, 209.5)	48.9 (24.7, 122.3)	73.7 (27.6, 276.2)	0.102
Operation time (hours)	8.0 (6.0, 9.0)	8.0 (7.0, 9.0)	8.0 (6.0, 9.0)	0.641
Intraoperative blood loss (ml)	450.0 (400.0, 600.0)	400.0 (400.0, 600.0)	500.0 (400.0, 725.0)	0.937
Tumor differentiation [n (%)]				0.618
Poor	65 (36.7)	18 (34.0)	47 (37.9)	
Moderate & high	112 (63.3)	35 (66.0)	77 (62.1)	
TNM stage [n (%)]				0.357
I-II	154 (87.0)	48 (90.6)	106 (85.5)	
III	23 (13.0)	5 (9.4)	18 (14.5)	
T stage [n (%)]				0.341
T1-T2	27 (15.3)	6 (11.3)	21 (16.9)	
T3	150 (84.7)	47 (88.7)	103 (83.1)	
Portal vein system invasion [n (%)]				0.996
Yes	15 (8.5)	5 (9.4)	10 (8.1)	
No	162 (91.5)	48 (90.6)	114 (91.9)	
Lymph nodes metastasis [n (%)]				0.496
Yes	77 (43.5)	21 (39.6)	56 (45.2)	
No	100 (56.5)	32 (60.4)	68 (54.8)	
Resection Margin [n (%)]				0.242
R0	169 (95.5)	49 (92.5)	120 (96.8)	
R1	8 (4.5)	4 (7.5)	4 (3.2)	
Postoperative chemotherapy [n (%)]				0.301
Yes	94 (53.1)	25 (47.2)	69 (55.6)	
No	83 (46.9)	28 (52.8)	55 (44.4)	
Postoperative complications [n (%)]				0.464
Yes	63 (35.6)	21 (39.6)	42 (33.9)	
No	114 (64.4)	32 (60.4)	82 (66.1)	
Pancreatic fistula [n (%)]	43 (24.3)	15 (28.3)	28 (22.6)	0.416
Delayed gastric emptying [n (%)]	17 (9.6)	7 (13.2)	10 (8.1)	0.288
Abdominal infection [n (%)]	26 (14.7)	7 (13.2)	19 (15.3)	0.716
Abdominal hemorrhage [n (%)]	16 (9.0)	3 (5.7)	13 (10.5)	0.399
Gastrointestinal bleeding [n (%)]	4 (2.3)	1 (1.9)	3 (2.4)	1.000

(dNLR, derived neutrophil-to-lymphocyte ratio; BMI, body mass index; WBC, white blood cell; NE, neutrophil; Hb, hemoglobin; PLT, platelet; ALB, albumin; TB, total bilirubin; CEA, caricno-embryonic antigen; CA19-9, carbohydrate antigen 19-9).

Following preoperative evaluation to exclude surgical contraindications, all patients underwent curative pancreaticoduodenectomy at our center. Patients identified intraoperatively as having portal venous invasion underwent concurrent vascular resection with end-to-end anastomosis reconstruction. Median operative time was 8.0 hours, and median intraoperative blood loss was 450.0 mL. Postoperative pathology confirmed dCCA (adenocarcinoma) in all patients. Portal vein invasion was present in 15 patients (8.5%), and lymph node metastasis was identified in 77 patients (43.5%). Postoperative complications occurred in 63 patients, yielding an overall complication rate of 35.6%. Details of major complications are presented in **Table 1**.

Patient follow-up continued until January 2025, with a median overall survival (OS) of 30 months. The 1-, 3-, and 5-year OS rates were 80.2%, 39.9%, and 30.1%, respectively. Median recurrence-free survival (RFS) was 26 months, with 1-, 3-, and 5-year RFS rates of 70.5%, 44.8%, and 36.1%, respectively.

### Patient stratification by preoperative dNLR and prognostic evaluation

**Figure 1:**
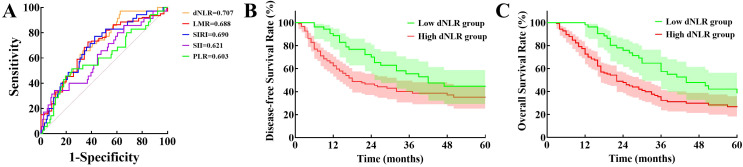
Evaluation of prognostic value of dNLR: **(A)** Comparison of receiver operator characteristic curves for derived neutrophil-to-lymphocyte ratio, lymphocyte-to-monocyte ratio, systemic inflammatory response index, systemic immune-inflammation index, and platelet-to-lymphocyte ratio in predicting postoperative prognosis. **(B)** Disease-free survival between low and high derived neutrophil-to-lymphocyte ratio groups. **(C)** Overall survival between the two groups. (dNLR, Derived neutrophil-to-lymphocyte ratio; LMR, Lymphocyte-to-monocyte ratio; SIRI, Systemic inflammatory response index; SII, Systemic immune-inflammation index; PLR, Platelet-to-lymphocyte ratio).

To evaluate the prognostic value of preoperative dNLR in dCCA, we generated a ROC curve correlating dNLR with 1-year postoperative survival. The AUC for dNLR was 0.707 (95% *CI*: 0.618–0.796), surpassing other previously reported inflammatory indices for dCCA prognosis, including the lymphocyte-to-monocyte ratio (LMR; 0.688, 95% *CI*: 0.591–0.785), platelet-to-lymphocyte ratio (PLR; 0.603, 95% *CI*: 0.492–0.714), systemic inflammatory response index (SIRI; 0.690, 95% *CI*: 0.595–0.786), and systemic immune-inflammation index (SII; 0.621, 95% *CI*: 0.514–0.728; **Figure 1A**). Based on the ROC analysis, the optimal dNLR cutoff value was determined to be 1.60, yielding a sensitivity of 97.1% and specificity of 37.4%.

Patients were subsequently categorized into low-dNLR (≤ 1.60, n = 53) and high-dNLR (> 1.60, n=124) groups. Comparison of perioperative characteristics and postoperative complications between groups is shown in **Table 1**. Preoperative total leukocyte counts, neutrophil counts, PLR, SIRI, SII, total bilirubin, and carcinoembryonic antigen (CEA) levels were significantly lower, whereas preoperative lymphocyte counts and LMR were significantly higher in the low-dNLR group compared to the high-dNLR group (*p <* 0.05). There were no significant differences between the groups regarding other baseline parameters, intraoperative characteristics, or postoperative complications (*p >* 0.05).

Median RFS time was significantly longer in the low-dNLR group (41 months) compared to the high-dNLR group (17 months), with 1-, 3-, and 5-year RFS rates of 88.7%, 55.5%, and 44.6% versus 62.4%, 40.2%, and 35.1%, respectively (χ² = 5.583, *p* = 0.018; **Figure 1B**). Median OS was also significantly greater in the low-dNLR group (44 months) than in the high-dNLR group (22 months), with corresponding 1-, 3-, and 5-year OS rates of 98.1%, 57.5%, and 38.8% compared to 72.5%, 32.3%, and 26.7% (χ² = 15.257, *p* < 0.001; **Figure 1C**).

### Independent risk factors for tumor recurrence after surgery for dCCA

Univariate analysis was conducted using preoperative clinical data, intraoperative data, postoperative pathology findings, and recovery data as independent variables, with RFS as the dependent variable. Results indicated that preoperative dNLR, CEA, CA19–9 levels, tumor differentiation, TNM stage, T stage, portal vein invasion, and lymph node metastasis were potential risk factors significantly associated with recurrence (*p* < 0.05; **Table 2**). Multivariate Cox regression analysis confirmed that a preoperative dNLR > 1.60 independently predicted postoperative tumor recurrence (*RR* = 1.735, 95% *CI*: 1.076 – 2.796). Additionally, T stage > T2, portal vein invasion, and lymph node metastasis were identified as independent predictors of postoperative recurrence in dCCA patients (**Table 3**).

**Table 2:** Univariate analysis of postoperative tumor recurrence in dCCA patients.

Variables	Number (*n*=177)	1-year DFS rate (%)	3-year DFS rate (%)	*χ* ^2^ Value	*P* Value
Gender				0.083	0.774
Male	89	67.0	46.4		
Female	88	75.6	42.4		
Age (years)				0.466	0.495
≤ 60	63	69.0	40.1		
> 60	114	71.0	46.4		
BMI (kg/m^2^)				2.704	0.100
≤ 24.92	115	67.9	40.9		
> 24.92	62	75.3	52.1		
Preoperative WBC (×10^9^/L)				1.040	0.308
≤ 5.50	67	77.2	46.9		
> 5.50	110	66.4	43.6		
Preoperative NE (×10^9^/L)				1.066	0.302
≤ 4.14	102	75.0	46.0		
> 4.14	75	64.3	40.5		
Preoperative LYM (×10^9^/L)				2.500	0.114
≤ 1.15	55	55.4	45.1		
> 1.15	122	77.3	45.8		
Preoperative dNLR				5.583	0.018*
≤ 1.60	53	88.7	55.5		
> 1.60	124	62.4	40.2		
Preoperative LMR				3.452	0.063
≤ 2.64	61	54.9	46.3		
> 2.64	116	78.7	45.4		
Preoperative SIRI				1.932	0.165
≤ 1.22	89	79.3	45.5		
> 1.22	88	61.5	44.9		
Preoperative SII				1.684	0.194
≤ 1411.04	150	74.7	44.5		
> 1411.04	27	46.7	40.8		
Preoperative PLR				0.888	0.346
≤ 225.88	125	76.1	45.2		
> 225.88	52	57.0	43.6		
Preoperative ALB (g/L)				3.637	0.056
≤ 36.65	80	64.3	38.0		
> 36.65	97	75.5	50.5		
Preoperative TB (μmol/L)				3.202	0.074
≤ 51.05	60	79.6	48.9		
> 51.05	117	65.8	42.9		
Preoperative CEA (ng/ml)				5.305	0.021*
≤ 2.55	130	74.1	50.2		
> 2.55	47	60.1	30.1		
Preoperative CA19-9 (U/ml)				4.380	0.036*
≤ 40.55	65	81.5	54.5		
> 40.55	112	63.7	38.5		
Operation time (hours)				1.069	0.301
≤ 8	117	69.5	43.5		
> 8	60	72.2	47.3		
Intra-operative blood loss (ml)				2.132	0.144
≤ 800	157	73.2	46.8		
> 800	20	50.0	28.1		
Tumor differentiation				10.579	0.001*
Poor	65	47.9	35.9		
Moderate & High	112	83.5	50.3		
T stage				9.827	0.002*
T1&T2	27	96.2	70.1		
T3	150	65,8	40.1		
Lymph node metastasis				32.593	<0.001*
Yes	77	54.0	25.8		
No	100	83.4	59.7		
Portal vein system invasion				10.948	0.001*
Yes	15	53.3	9.3		
No	162	72.1	48.0		
Resection margin				3.357	0.067
R0	169	71.5	46.7		
R1	8	50.0	12.5		
TNM Stage				19.026	<0.001*
I & II	154	75.4	49.2		
III	23	36.3	15.5		
Postoperative complications				0.142	0.707
Yes	63	70.8	44.2		
No	114	69.9	46.0		
Postoperative chemotherapy				1.805	0.179
Yes	94	69.6	36.7		
No	83	71.3	32.4		

(DFS, Disease-free survival; BMI, body mass index; WBC, white blood cell; NEUT, neutrophil; LYM, lymphocyte; dNLR, derived neutrophil-to-lymphocyte ratio; LMR, lymphocyte-to-monocyte ratio; SIRI, systemic inflammation response index; SII, systemic immune-inflammation index; PLR, platelet-to-lymphocyte ratio; ALB, albumin; TB, total bilirubin; CEA, caricno-embryonic antigen; CA19-9, carbohydrate antigen 19-9; RR, Relative risk; CI, Confidence interval; *: *P*<0.05).

**Table 3:** Multivariate analysis of postoperative tumor recurrence in dCCA patients.

Variables	*RR* Value	95% *CI*	*P* Value
Preoperative dNLR > 1.60	1.735	1.076-2.796	0.024*
Preoperative CEA > 2.55 ng/ml	1.239	0.787-1.949	0.355
Preoperative CA19-9 > 40.55 U/ml	1.311	0.834-2.063	0.241
Low tumor differentiation degree	1.420	0.932-2.163	0.103
TNM stage > II	1.006	0.542-1.866	0.985
T stage > 2	2.952	1.399-6.227	0.004*
Portal vein system invasion	2.720	1.488-4.975	0.001*
Lymph node metastasis	2.698	1.707-4.262	<0.001*

(dNLR, derived neutrophil-to-lymphocyte ratio; CEA, caricno-embryonic antigen; CA19-9, carbohydrate antigen 19-9; RR, Relative risk; CI, Confidence interval; *: *P*<0.05).

### Independent risk factors for long-term overall survival in dCCA

Univariate analysis using preoperative clinical data, intraoperative data, postoperative pathological findings, and postoperative recovery data identified preoperative lymphocyte count, dNLR, LMR, SIRI, albumin, total bilirubin, CEA, CA19-9, tumor differentiation, TNM stage, T stage, portal vein invasion, and lymph node metastasis as significant risk factors associated with overall survival (*p* < 0.05; **Table 4**). Multivariate Cox regression confirmed that a preoperative dNLR > 1.60 was an independent predictor of decreased overall survival (*RR* = 1.777, 95% *CI*: 1.081 – 2.922). Additionally, preoperative total bilirubin > 51.05 µmol/L, CA19-9 > 40.55 mmol/L, T stage > T2, portal vein invasion, and lymph node metastasis emerged as significant independent risk factors for poor overall survival in dCCA (**Table 5**).

**Table 4:** Univariate analysis of postoperative long-term survival in dCCA patients.

Variables	Number(*n*=177)	1-year OS rate (%)	3-year OS rate (%)	*χ* ^2^ Value	*P* Value
Gender				0.294	0.587
Male	89	79.2	41.3		
Female	88	80.3	36.9		
Age (years)				0.004	0.947
≤ 60	63	80.4	38.7		
> 60	114	79.3	40.4		
BMI (kg/m^2^)				3.003	0.083
≤ 24.92	115	75.6	38.0		
> 24.92	62	88.7	43.8		
Preoperative WBC (×10^9^/L)				2.676	0.102
≤ 5.50	67	85.1	45.8		
> 5.50	110	77.2	36.3		
Preoperative NE (×10^9^/L)				1.913	0.167
≤ 4.14	102	86.2	41.8		
> 4.14	75	72.0	37.4		
Preoperative LYM (×10^9^/L)				4.140	0.042*
≤ 1.15	55	67.1	34.1		
> 1.15	122	86.1	43.7		
Preoperative dNLR				9.386	0.002*
≤ 1.60	53	98.1	57.5		
> 1.60	124	72.5	32.3		
Preoperative LMR				9.108	0.003*
≤ 2.64	61	63.7	31.7		
> 2.64	116	88.8	44.3		
Preoperative SII				1.921	0.166
≤ 1411.04	150	84.6	40.0		
> 1411.04	27	55.6	39.9		
Preoperative SIRI				7.039	0.008*
≤ 1.22	89	91.0	46.3		
> 1.22	88	69.2	33.4		
Preoperative PLR				1.370	0.242
≤ 225.88	125	86.4	41.9		
> 225.88	52	65.4	35.3		
Preoperative ALB (g/L)				6.893	0.009*
≤ 36.65	80	73.8	32.7		
> 36.65	97	85.6	46.3		
Preoperative TB (μmol/L)				9.182	0.002*
≤ 51.05	60	86.6	53.5		
> 51.05	117	75.2	33.0		
Preoperative CEA (ng/ml)				4.802	0.028*
≤ 2.55	130	83.8	44.6		
> 2.55	47	70.1	28.4		
Preoperative CA19-9 (U/ml)				14.493	< 0.001*
≤ 40.55	65	95.4	57.8		
> 40.55	112	71.4	29.5		
Operation time (hours)				1.073	0.300
≤ 8	117	78.6	38.0		
> 8	60	83.2	44.2		
Intra-operative blood loss (ml)				1.306	0.253
≤ 800	157	80.9	42.1		
> 800	20	75.0	21.7		
Tumor differentiation				8.738	0.003*
Poor	65	64.6	33.0		
Moderate & High	112	89.3	44.5		
T stage				9.173	0.002*
T1&T2	27	92.6	67.8		
T3	150	78.0	34.6		
Lymph node metastasis				21.799	<0.001*
Yes	77	74.0	23.0		
No	100	85.0	52.6		
Portal vein system invasion				8.590	0.003*
Yes	15	66.7	14.8		
No	162	81.5	42.3		
Resection margin				1.349	0.246
R0	169	80.4	40.8		
R1	8	75.0	25.0		
TNM Stage				12.225	<0.001*
I & II	154	82.5	44.0		
III	23	65.2	8.8		
Postoperative complications				0.771	0.380
Yes	63	82.5	34.2		
No	114	78.9	43.0		
Postoperative chemotherapy				0.141	0.707
Yes	94	81.8	40.0		
No	83	78.7	39.8		

(OS, Overall survival; BMI, body mass index; WBC, white blood cell; NEUT, neutrophil; LYM, lymphocyte; dNLR, derived neutrophil-to-lymphocyte ratio; LMR, lymphocyte-to-monocyte ratio; SIRI, systemic inflammation response index; SII, systemic immune-inflammation index; PLR, platelet-to-lymphocyte ratio; ALB, albumin; TB, total bilirubin; CEA, caricno-embryonic antigen; CA19-9, carbohydrate antigen 19-9; RR, Relative risk; CI, Confidence interval; *: *P*<0.05).

**Table 5:** Multivariate analysis of postoperative long-term survival in dCCA patients.

Variables	*RR* Value	95% *CI*	*P* Value
Preoperative LYM ≤ 1.15×10^9^/L	1.117	0.701-1.780	0.641
Preoperative dNLR > 1.60	1.777	1.081-2.922	0.024*
Preoperative LMR ≤ 2.64	1.035	0.561-1.912	0.911
Preoperative SIRI > 1.22	1.001	0.555-1.806	0.997
Preoperative ALB ≤ 36.65 g/L	1.177	0.783-1.770	0.434
Preoperative TB > 51.05 μmol/L	1.615	1.015-2.571	0.043*
Preoperative CEA > 2.55 ng/ml	1.211	0.797-1.841	0.369
Preoperative CA19-9 > 40.55 U/ml	2.007	1.277-3.155	0.003*
Low tumor differentiation degree	1.296	0.869-1.932	0.203
TNM stage > II	1.046	0.547-1.999	0.892
T stage > 2	2.820	1.435-5.543	0.003*
Portal vein system invasion	2.090	1.129-3.871	0.019*
Lymph node metastasis	1.995	1.272-3.131	0.003*

(LYM, lymphocyte; dNLR, derived neutrophil-to-lymphocyte ratio; LMR, lymphocyte-to-monocyte ratio; SIRI, systemic inflammation response index; ALB, albumin; TB, total bilirubin; CEA, caricno-embryonic antigen; CA19-9, carbohydrate antigen 19-9; RR, Relative risk; CI, Confidence interval; *: *P*<0.05)

### Machine learning–based prognostic feature selection for overall survival

**Figure 2:**
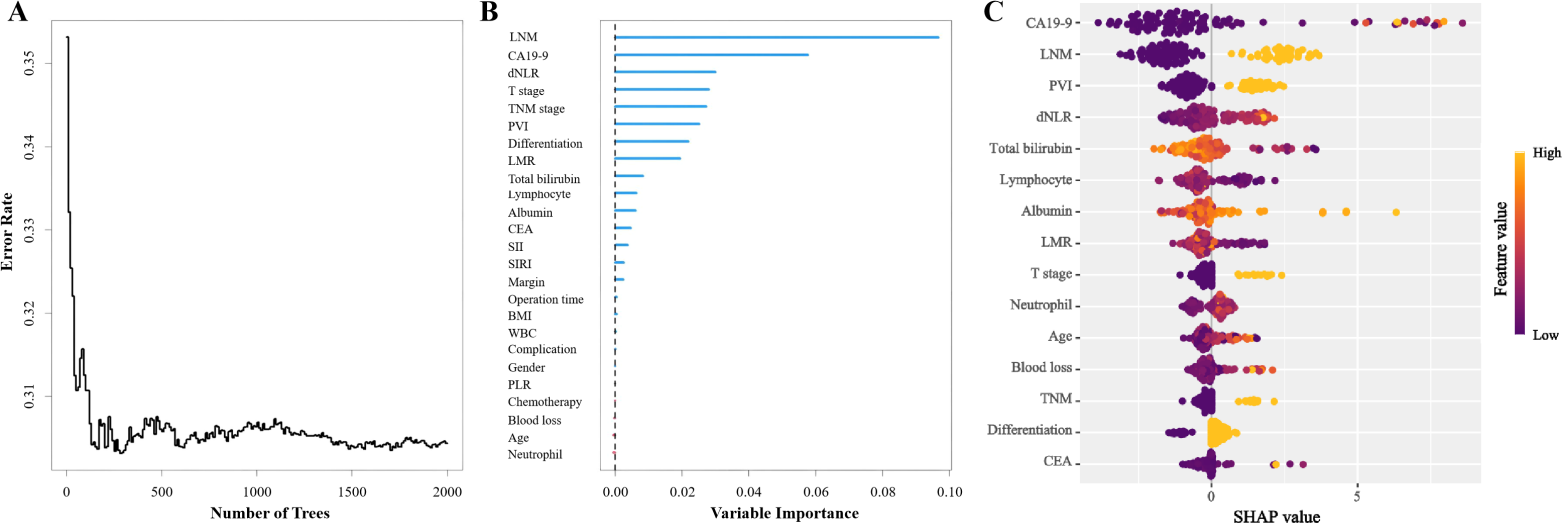
Random Survival Forest model for patients with distal cholangiocarcinoma: **(A)** Change of error rate with number of trees. **(B)** Variable importance of Random Survival Forest model. **(C)** SHapley Additive exPlanations of Random Survival Forest model. (LNM, Lymph node metastasis; CA19-9, Carbohydrate antigen 19-9; dNLR, Derived neutrophil-to-lymphocyte ratio; PVI, Portal vein invasion; LMR, Lymphocyte-to-monocyte ratio; CEA, Carcino-embryonic antigen; SII, Systemic immune-inflammation index; SIRI, Systemic inflammatory response index; BMI, Body mass index; WBC, White blood cell; PLR, Platelet-to-lymphocyte ratio).

Machine learning methods were applied to jointly identify prognostic risk factors associated with OS. RSF was first used to assess the importance of all candidate variables. Variables were ranked according to their importance scores, and features showing the highest relevance to prognosis were preliminarily selected (**Figure 2**).

**Figure 3:**
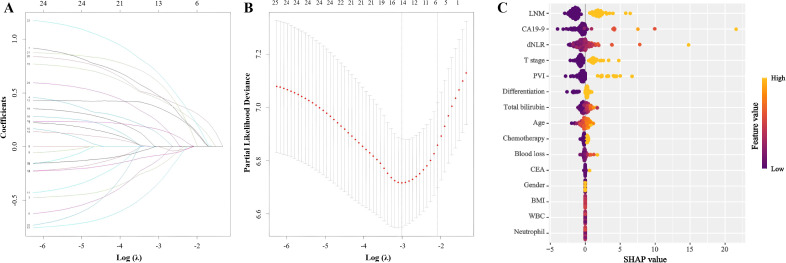
Lasso-Cox regression for variable selection. **(A)** Lasso coefficient profiles of the 25 variables. The x-axis represents the log-transformed lambda (λ), and the y-axis shows the coefficients. **(B)** Ten-fold cross-validation for tuning parameter selection in the Lasso-Cox model. The x-axis represents log(λ), and the y-axis shows the partial likelihood deviance. **(C)** SHapley Additive exPlanations of Lasso-Cox regression model. (LNM, Lymph node metastasis; CA19-9, Carbohydrate antigen 19-9; dNLR, Derived neutrophil-to-lymphocyte ratio; PVI, Portal vein invasion; CEA, Carcino-embryonic antigen; BMI, Body mass index; WBC, White blood cell).

Subsequently, least absolute shrinkage and selection operator–penalized Cox regression (LASSO-Cox) was employed for regularized regression analysis. Among the variables, dNLR, T stage, and lymph node metastasis exhibited the highest absolute coefficients (**Figure 3**).

**Figure 4:**
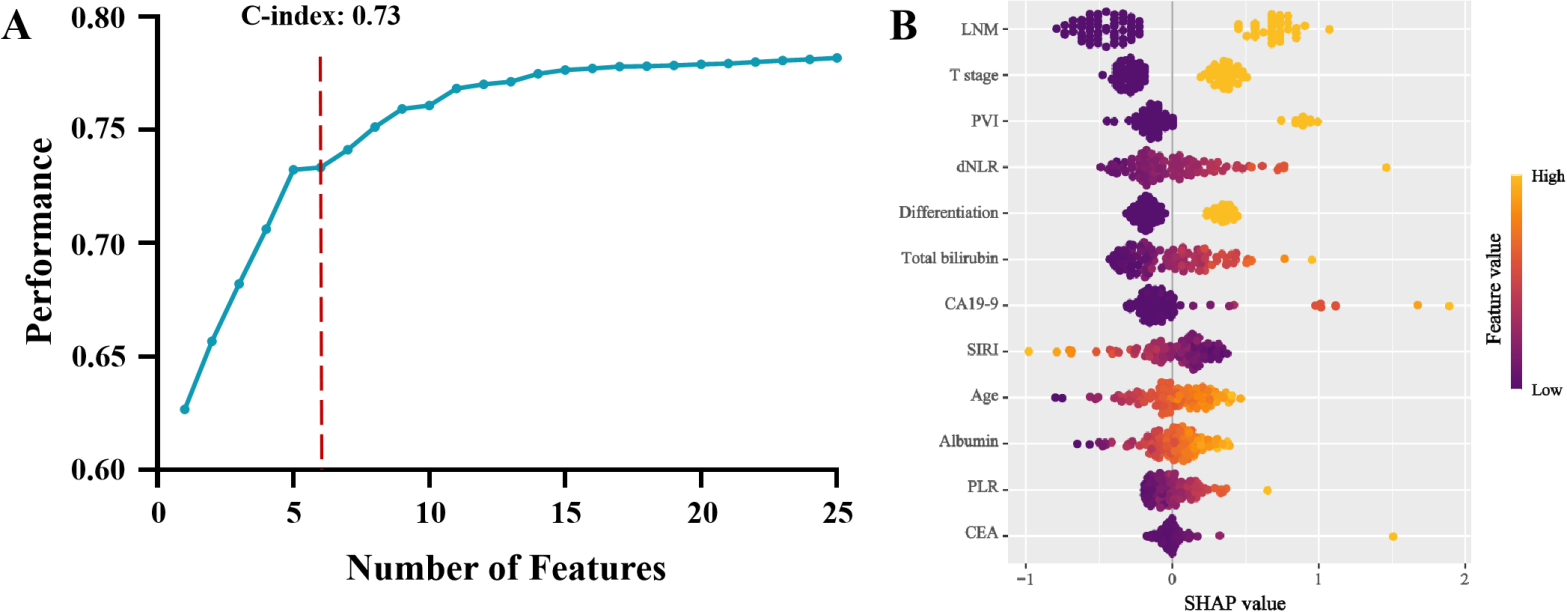
Recursive feature elimination algorithm model and its variable importance: **(A)** Predictive performance of different feature subsets. **(B)** SHapley Additive exPlanations of Recursive feature elimination algorithm model (LNM, Lymph node metastasis; PVI, Portal vein invasion; dNLR, Derived neutrophil-to-lymphocyte ratio; CA19-9, Carbohydrate antigen 19-9; SIRI, Systemic inflammatory response index; PLR, Platelet-to-lymphocyte ratio; CEA, Carcino-embryonic antigen).

Recursive feature elimination was then conducted to evaluate the predictive performance of different variable subsets. The optimal subset was determined based on cross-validation, and the top six variables were identified as the most suitable: dNLR, total bilirubin, CA19-9, lymph node metastasis, portal vein invasion, and T stage (**Figure 4**). These six factors were further incorporated into a multivariate Cox regression model. Integrating the results from all three selection strategies, the final model was built and validated based on this selected feature set.

### Establishment and validation of a prognostic model for dCCA based on dNLR

**Figure 5:**
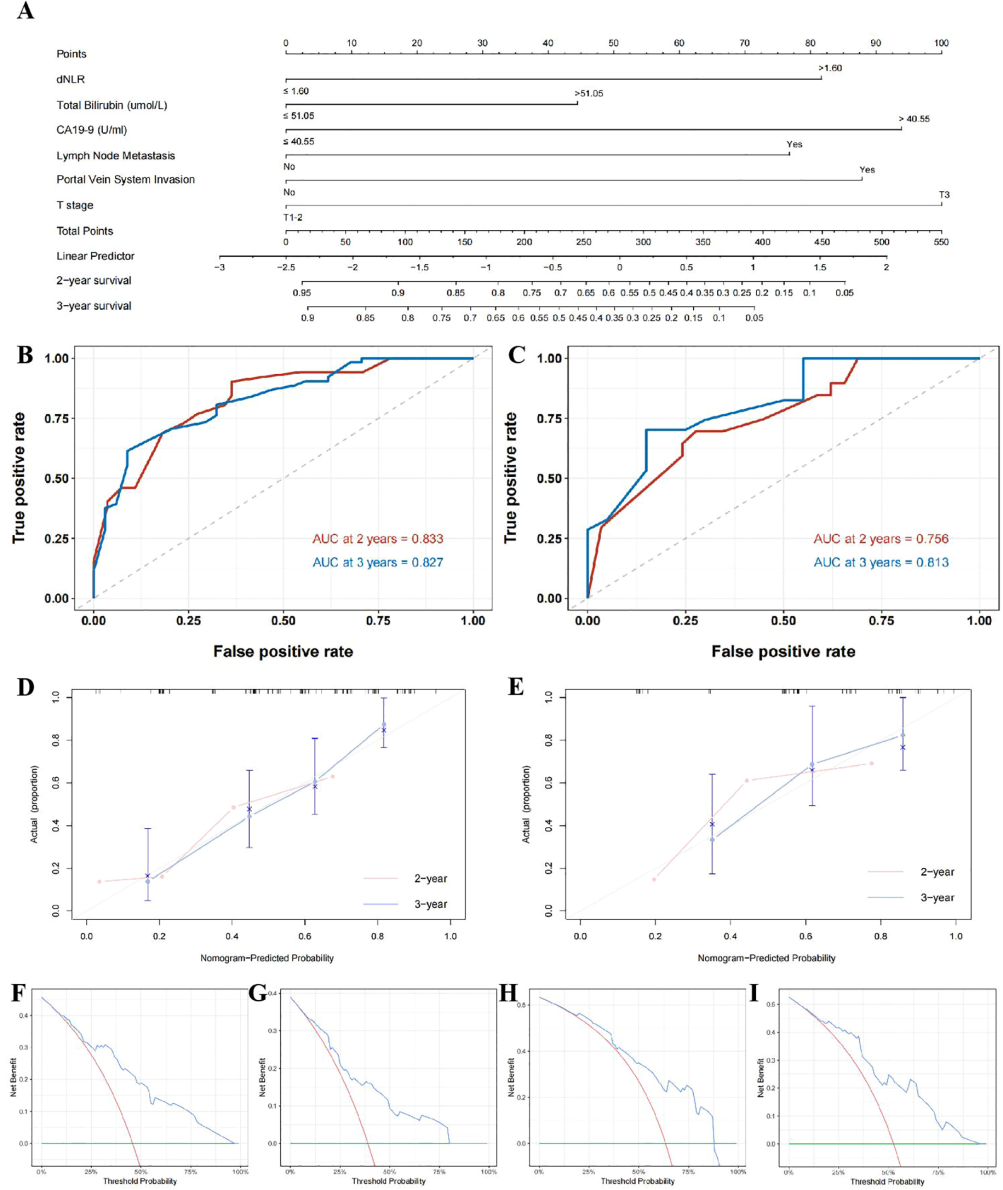
Nomogram based on dNLR for prognostic prediction in distal cholangiocarcinoma. **(A)**; time-dependent ROC curves for predicting 2- and 3-year postoperative survival in the training cohort **(B)** and validation cohort **(C)**; calibration curves for the nomogram in the training cohort **(D)** and validation cohort **(E)**; decision curve analysis (DCA) for predicting 2-year survival in the training cohort **(F)** and validation cohort **(G)**, and 3-year survival in the training cohort **(H)** and the validation cohort **(I)**.

To further enhance the prognostic utility of dNLR for dCCA outcomes, enrolled patients were randomly divided into training (70%) and validation (30%) sets. A predictive nomogram incorporating independent risk factors identified through multivariate Cox regression was constructed (**Figure 5A**). ROC analyses of this nomogram showed favorable performance in predicting 2- and 3-year postoperative survival in the training set (AUC: 0.833, 0.756) and validation set (AUC: 0.827, 0.813; **Figures 5B, C**). As observed in the calibration curve (**Figures 5D, E**), the points generally followed the diagonal line across most of the probability range in both training set and validation set, demonstrating a reasonably good calibration performance in postoperative 2- and 3-year prognosis prediction in both training and validation set. DCA curves demonstrated this model provided net benefits in predicting postoperative 2- and 3-year survival outcomes in training and validation set under the threshold probability of 5-96%, 9-87% and 5-79%, 9-95%, respectively, further demonstrating the clinical applicability of this model in predicting postoperative prognosis of dCCA patients (**Figures 5F–I**).

## Discussion

dCCA is a relatively uncommon malignancy associated with poor long-term survival outcomes. Early prediction of postoperative survival in dCCA can guide clinical decision-making, optimize follow-up intervals, and facilitate earlier detection of recurrence or metastasis, thus potentially improving patient prognosis. In this study, we first reported the predictive value of the dNLR for postoperative prognosis in dCCA, confirming that an elevated dNLR is an independent adverse prognostic factor. Moreover, due to the unsatisfying specificity of dNLR in predicting postoperative prognosis, we further integrated dNLR with other independent risk factors into a Cox regression model to enhance its predictive accuracy, providing a robust and clinically applicable model to more precisely estimate prognosis in patients with dCCA. In clinical practice, both dNLR and proposed prediction model may better guide clinical decision making. Patients with low dNLR and risk points may benefit from surgical treatment and achieve long-term survival, and should consider upfront surgery firstly. For those with high dNLR and risk points, clinicians should arrange more frequent follow-up schedule and earlier implementation of adjuvant therapy if necessary to prolong their postoperative survival time.

As an emerging inflammatory biomarker, dNLR includes only peripheral leukocyte and neutrophil counts, rendering it convenient for clinical computation and application. Previously, dNLR was primarily identified as a valuable predictor of response to immune checkpoint inhibitors in multiple malignancies (18, 19). Additionally, several studies have validated high preoperative dNLR as an independent risk factor for poor postoperative prognosis in cancer patients. Deng et al., retrospectively analyzing 389 gastric cancer patients who underwent radical resection, reported an AUC of 0.683 for dNLR predicting postoperative survival, identifying dNLR >1.85 as significantly associated with poor OS (20). Similarly, Absenger et al. observed prolonged DFS (132 months) and OS time (147 months) in colorectal cancer patients with low dNLR compared to those with elevated levels (21). Zhang et al. further demonstrated in 231 iCCA patients that dNLR ≥1.5 was significantly correlated with adverse postoperative outcomes (17). Consistent with these findings, our study extended the clinical applicability of dNLR, establishing it as an independent predictor of postoperative prognosis in dCCA patients. Furthermore, to comprehensively assess the predictive performance of dNLR, we compared its prognostic AUC (0.707) with other previously reported inflammatory biomarkers including LMR, SII, PLR, and SIRI, revealing a superior prognostic value of dNLR. It was also identified as an important risk factor for postoperative prognosis according to machine-learning models and Cox regression models, underscoring its efficacy and clinical utility in predicting postoperative outcomes in dCCA patients.

Current evidence suggests that the poor prognosis associated with high dNLR primarily results from elevated peripheral neutrophil counts. Neutrophils are central components of inflammatory responses and have been reported to promote tumor progression through multiple pathways. First, neutrophils generate reactive oxygen species (ROS) inducing tissue and DNA damage, thus facilitating carcinogenesis, and further enhancing tumor metastasis through modulation of cytokines such as IL-1β (22, 23). Additionally, neutrophils secrete cytokines including TGF-β, VEGF, OSM, IL-10, and HGF, which directly promote tumor angiogenesis and tumor progression (24). Moreover, under the influence of tumor cells, neutrophils can form neutrophil extracellular traps (NETs), contributing further to tumor progression (25). Recent studies have specifically explored neutrophil-driven progression in cholangiocarcinoma. Zhou et al. reported that tumor-associated neutrophils interacting with macrophages promote the secretion of OSM in intrahepatic cholangiocarcinoma, subsequently activating STAT3 signaling and facilitating tumor growth (26). Yoshimoto et al. observed neutrophil extracellular traps enhancing metastatic potential in intrahepatic cholangiocarcinoma cells, with Zhang et al. further elucidating that NET-DNA activation of ITGAV/NFκB signaling increased tumor proliferation, activation, metastasis, and adversely influenced prognosis (27, 28). These findings collectively suggest that neutrophils infiltrating cholangiocarcinoma predominantly exert pro-tumorigenic effects. In our cohort, elevated neutrophil counts observed in high-dNLR patients align with previously reported correlations between peripheral neutrophilia and local neutrophil infiltration in cholangiocarcinoma. Hence, we speculate that elevated dNLR could reflect increased local neutrophil infiltration, thereby accelerating tumor progression and metastasis and ultimately adversely affecting patient outcomes (29).

In addition to elevated neutrophil counts, our cohort demonstrated significantly reduced lymphocyte counts in patients with high dNLR. Lymphocytes are the primary mediators of antitumor immunity, and lower lymphocyte counts or proportions directly reflect impaired antitumor immune responses, correlating with poor prognosis across numerous malignancies (30–32). In cholangiocarcinoma, lymphocytes—particularly CD8^+^ T cells—play a critical prognostic role. Kitano et al. reported that reduced local CD8^+^ T-cell infiltration was associated with significantly poorer overall survival in extrahepatic CCA patients; similarly, Kim et al. demonstrated that patients with higher CD8^+^ T-cell infiltration (≥100 cells/high-power field) exhibited improved OS time and DFS time (49.7 and 23.3 months, respectively) compared to controls (33, 34). Beyond CD8^+^ T cells, atypical T-cell subsets also exhibit potent antitumor activity. Zimmer et al. identified mucosal-associated invariant T (MAIT) cells in iCCA, correlating increased local MAIT cell infiltration with substantially improved survival (median: 59.5 months) (35). Likewise, increased counts of natural killer (NK) cells with recognized antitumor properties have been positively correlated with improved survival in CCA patients (36). These data reinforce that lymphocyte counts and proportions significantly influence recurrence and prognosis in CCA. Consequently, the combination of increased neutrophils and decreased lymphocytes, characteristic of high-dNLR patients, likely contributes significantly to their inferior prognosis.

Currently, adjuvant chemotherapy constitutes an essential component in CCA management, significantly influencing patient prognosis. Previous studies have demonstrated the utility of dNLR in predicting chemotherapy responsiveness, with high-dNLR patients exhibiting superior sensitivity to gemcitabine-cisplatin/oxaliplatin regimens compared to those with low dNLR (15, 16). Given that gemcitabine and platinum-based regimens remain standard adjuvant treatments for CCA, we hypothesize that high-dNLR patients may particularly benefit from postoperative chemotherapy, potentially improving survival outcomes, and propose dNLR as a predictive marker for chemotherapy response. However, due to incomplete chemotherapy records and regimen heterogeneity in our cohort, we could not definitively analyze the predictive value of dNLR for chemotherapy response—an area warranting future research.

Our study also identified elevated total bilirubin levels and the classical tumor marker CA19–9 as independent risk factors associated with poor long-term prognosis in patients with dCCA. CA19–9 is widely utilized as a serological biomarker for both the clinical diagnosis and prognostic evaluation of CCA. Tella et al. (37) reviewed data from the United States National Cancer Database (NCDB), encompassing 2,100 patients with extrahepatic CCA, and found that CA19–9 levels were elevated in nearly 1,500 patients, accounting for over 70% of the cohort. Notably, patients with elevated CA19–9 exhibited a significantly shorter median survival compared to those with normal CA19–9 levels (8.5 months vs. 16.0 months, *p* < 0.01). Furthermore, CA19–9 was identified as an independent prognostic factor for long-term survival (*RR* = 1.72, 95% *CI*: 1.46–2.02). Interestingly, subsequent studies have highlighted that in cases of dCCA and pancreatic head carcinoma, obstructive jaundice caused by tumor-induced biliary obstruction can lead to an abnormal elevation of both bilirubin and CA19–9 levels. Such elevations may not accurately reflect the true tumor burden or predict long-term outcomes, thereby confounding prognostic assessments. In response to this challenge, innovative approaches have been proposed, such as employing the ratio of CA19–9 to bilirubin to mitigate the influence of inflammation and biliary obstruction on biomarker interpretation (38–40).

Consistent with these findings, our study also demonstrated that bilirubin and CA19–9 exert independent, non-collinear effects on the postoperative prognosis of dCCA patients. This reinforces the concept that both markers should be carefully interpreted as distinct prognostic indicators. In future clinical applications, these findings may enable clinicians to more precisely evaluate and intervene in the postoperative management of dCCA patients, ultimately contributing to improved long-term outcomes in this challenging population.

This study has several limitations. First, as a single-center retrospective study, selection bias, including sample selection and data recording, was unavoidable, limiting the reliability and promotion value of our result. Prospective multicenter studies with larger sample sizes are necessary to confirm our conclusions. Second, although our predictive model incorporating dNLR underwent rigorous internal validation, external validation remains essential for broader applicability. Third, while we discussed possible reasons underlying dNLR’s predictive utility, our study did not conclusively elucidate underlying biological mechanisms, which warrant further investigation. Lastly, we did not assess the predictive role of dNLR for adjuvant treatment efficacy, thus restricting clinical applicability; future studies should further explore this aspect to comprehensively validate dNLR’s clinical utility.

## Conclusion

As a composite blood-based inflammatory markers, dNLR serves as a novel prognostic indicator for dCCA. CA19–9 levels, lymph node metastasis, portal vein invasion, and tumor differentiation were identified as independent prognostic factors affecting survival in dCCA patients as well. By employing our machine-learning-driven prognostic prediction model, clinicians can facilitate early risk stratification and targeted interventions, ultimately contributing to improved clinical outcomes in patients with dCCA.

## Data Availability

The raw data supporting the conclusions of this article will be made available by the authors, without undue reservation.
